# Determination of minimum inhibitory and bactericidal concentrations of reduced graphene oxide–functionalized methylene blue for root canal disinfection: An in-vitro study

**DOI:** 10.12669/pjms.41.10.12576

**Published:** 2025-10

**Authors:** Rizwan Jouhar, Mohamad Syahrizal Halim, Sayed A. Quadri, Muhammad Adeel Ahmed

**Affiliations:** 1 Rizwan Jouhar, Department of Restorative Dental Sciences, College of Dentistry, King Faisal University, Al-Ahsa 31982, Saudi Arabia; 2 Mohamad Syahrizal Halim, Conservative Dentistry Unit, School of Dental Sciences, Health Campus, Universiti Sains Malaysia, Kota Bharu 16150, Kelantan, Malaysia; 3 Sayed A. Quadri, Division of Microbiology, Department of Biomedical Sciences, College of Medicine, King Faisal University, Al-Ahsa, Saudi Arabia; 4 Muhammad Adeel Ahmed, Department of Restorative Dental Sciences, College of Dentistry, King Faisal University, Al-Ahsa 31982, Saudi Arabia

**Keywords:** Antibacterial activity, *Enterococcus faecalis*, Graphene oxide, Methylene blue, Photodynamic therapy, MIC, MBC, Root canal disinfection

## Abstract

**Background and Objective:**

*Enterococcus faecalis (E. faecalis)* is a persistent endodontic bacterium that often resists standard antimicrobial treatments. Improving the effectiveness of methylene blue (MB) by combining it with reduced graphene oxide (MB-rGO) and activating it using laser light presents a potential alternative antibacterial approach. Therefore, this study evaluated and compared the antimicrobial activity of laser-activated MB and MB-rGO against *E. faecalis* by determining their minimum inhibitory and bactericidal concentration (MIC and MBC).

**Methodology:**

This in-vitro study was performed for a period of eight months i.e from June 2024 to January 2025 at Universiti Sains Malaysia and King Faisal University Al-Ahsa Saudi Arabia. The antimicrobial activity of MB and MB-rGO against *E. faecalis* ATCC 29212 was assessed using the broth microdilution method following CLSI guidelines. A standardized inoculum (5 × 10^5^ CFU/mL) was prepared in Mueller–Hinton broth. Stock solutions of MB and MB-rGO (800 µg/mL) were serially diluted to final concentrations ranging from 400 to 12.5 µg/mL. The test was conducted in 96-well microtiter plates, followed by photoactivation using a 660 nm diode laser for two minutes. Plates were incubated at 37°C for 24 hours. MIC was recorded as the lowest concentration without visible turbidity, while MBC was determined by subculturing from clear wells onto blood agar. Each test was performed three times independently under the same conditions to ensure reliability and consistency of the results.

**Results:**

Turbidity was observed at lower concentrations (12.5 and 25 µg/mL), while higher concentrations showed complete inhibition. Methylene blue (MB) demonstrated MIC and MBC values of 66.67 ± 28.87 µg/mL and 266.67 ± 115.47 µg/mL, respectively, whereas MB-rGO exhibited more consistent values of 50.00 ± 0.00 µg/mL and 200.00 ± 0.00 µg/mL. However, statistical analysis revealed no significant differences (p>0.05) between the groups.

**Conclusion:**

Methylene blue functionalized with reduced graphene oxide (MB-rGO) showed more consistent antibacterial activity under photoactivation, suggesting its potential as an enhanced antimicrobial agent against *E. faecalis*.

## INTRODUCTION

The long-term success of root canal treatment depends on effective disinfection of the canal and the subsequent maintenance of that disinfection through proper obturation and a hermetic seal.^1^,^2^ Achieving a bacteria-free canal is clinically challenging due to the complex anatomy of the root canal system. Variations such as lateral canals, apical ramifications, and fine dentinal tubules can hinder the penetration of disinfectants and promote bacterial colonization and biofilm formation.^2^,^3^ One of the major challenges clinicians face in eliminating bacteria from the canal is the presence of anaerobic microorganisms, particularly *E. faecalis*. This resilient bacterium can withstand harsh conditions, thrive in low-oxygen environments, and form persistent biofilms within the canal system.^4^,^5^ As a result, recent studies have increasingly focused on combining robust mechanical and chemical methods to achieve more effective canal disinfection.^6^-^8^

Antimicrobial photodynamic therapy (aPDT) has been introduced as an innovative disinfection process to adjunct the cleaning and shaping of root canals. In this method, a laser or LED light of a particular wavelength is used to activate a non-toxic photosensitizer in the root canal. Upon activation, the photosensitizers produce oxygen free radicals, which later on attack not only planktonic bacteria but also the biofilm cell wall, hence helping in achieving a sterile environment for a successful root canal therapy.^9^ Moreover, aPDT has the advantage that it has a localized mode of action, due to which it has no systemic side effects.^10^-^12^

Various photosensitizers have been investigated to enhance the efficacy of antimicrobial photodynamic therapy (aPDT), including phenothiazinium dyes (methylene blue, toluidine blue O), xanthene dyes (Rose Bengal, erythrosine), cyanine dyes (indocyanine green), and natural agents (curcumin, riboflavin). Among them, methylene blue remains the most widely used due to its early clinical approval, availability, and ease of application.^10^ Methylene blue (MB), also called methylthioninium chloride, is hydrophilic in nature with the absorption spectrum of 640 nm wavelength light, hence it produces free radicals with LED light or diode LASER of similar wavelength. It has wide antimicrobial properties and is found effective against bacteria and fungi.^13^

Graphene oxide (GO) is one of the most frequently employed nanoparticles in biomedical fields.^14^ Originating from grapheme, a two-dimensional, single-atom-thick sheet of carbon atoms arranged in a hexagonal pattern, GO has gained considerable research interest because of its remarkable antimicrobial properties. Its antibacterial effects result from multiple mechanisms, including the induction of oxidative stress, extraction of phospholipid molecules from bacterial membranes, physical disruption of the cell wall, trapping of microbial cells, and a self-killing effect.^14^ Moreover, GO serves as an effective carrier for drugs and biomolecules, improving their delivery, bioavailability, and overall antimicrobial performance of therapeutic agents.^15^

The working hypothesis of this study was that Methylene blue functionalized with reduced graphene oxide (MB-rGO), when activated by laser, exhibits significantly lower MIC and MBC values compared to methylene blue (MB) alone against *Enterococcus faecalis*, indicating enhanced antimicrobial efficacy. *E. faecalis* is a prevalent and persistent pathogen in endodontic infections, frequently resistant to conventional treatments. Functionalizing methylene blue (MB) with reduced graphene oxide (rGO) and applying photoactivation may improve its antibacterial effectiveness, offering a potentially improved treatment approach. Therefore, this study assessed and compared the antimicrobial activity of MB and MB-rGO against *E. faecalis* under photoactivation by examining their minimum inhibitory and bactericidal concentration (MIC & MBC).

## METHODOLOGY

This in-vitro study was performed for a period of eight months i.e, from June 2024 to January 2025.This study included the standard bacterial strain *Enterococcus faecalis* ATCC 29212, methylene blue (MB) and reduced graphene oxide–functionalized MB (MB-rGO) prepared using validated protocols, and test concentrations ranging from 12.5 µg/mL to 400 µg/mL. Only samples photoactivated using a 660 nm diode laser were evaluated, with experiments conducted in triplicate using freshly prepared bacterial suspensions in Mueller–Hinton broth under controlled aerobic incubation at 37°C. Samples were excluded if they involved unactivated photosensitizers, contaminated reagents, deviations from incubation conditions, or contamination observed in control wells.

### Ethical Approval:

It was obtained from King Faisal University (KFU-REC-2024-MAR-ETHICS2072; dated March 20, 2024) and Universiti Sains Malaysia (USM/JEPeM/KK/24030230; dated June 13, 2024).

### Preparation of the standardized inoculum:

The broth microdilution technique was employed following the Clinical and Laboratory Standards Institute (CLSI) guidelines.^16^ *Enterococcus faecalis* ATCC 29212 was chosen as the test strain due to its clinical importance and its standardized use in antimicrobial susceptibility assays.^17^

Microbial suspensions were prepared using the colony suspension method in Mueller–Hinton broth (MHB; Difco Laboratories, USA). Between three to five colonies of *E. faecalis* from a 24 hours culture grown on sheep blood agar (SBA) (5% defibrinated sheep blood; Watin-Biolife, Riyadh, Saudi Arabia) were suspended in MHB. The turbidity was adjusted to match a 0.5 McFarland standard, equivalent to roughly 1.5 × 10^8^ CFU/mL, and verified spectrophotometrically at 530 nm.^18^ The suspension was then diluted in sterile saline to achieve a final inoculum of 5 × 10^5^ CFU/mL.

### Preparation of test solutions:

**Fig.1:**
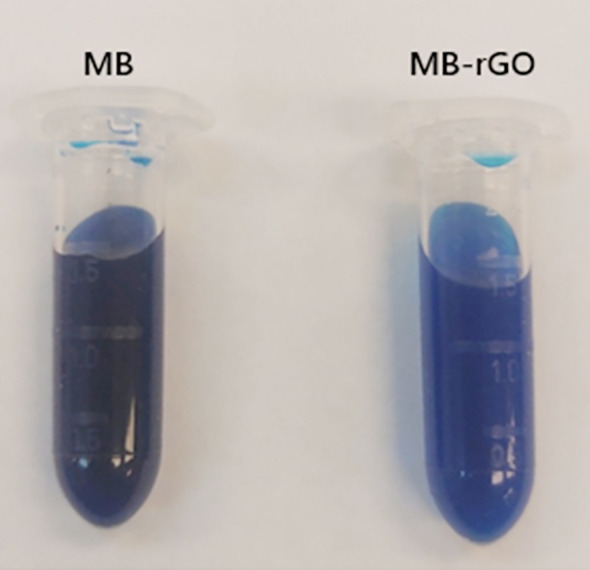
Stock Solutions of Methylene Blue (MB) and MB-Reduced Graphene Oxide (MB-rGO).

A stock solution at a concentration of 800 µg/mL was prepared in MHB (Difco Laboratories, USA) (Fig.1). Based on prior functionalization protocols^19^, the final concentrations used for each reagent were 400, 200, 100, 50, 25, and 12.5 µg/mL.

### Testing Protocol:

**Fig. 2:**
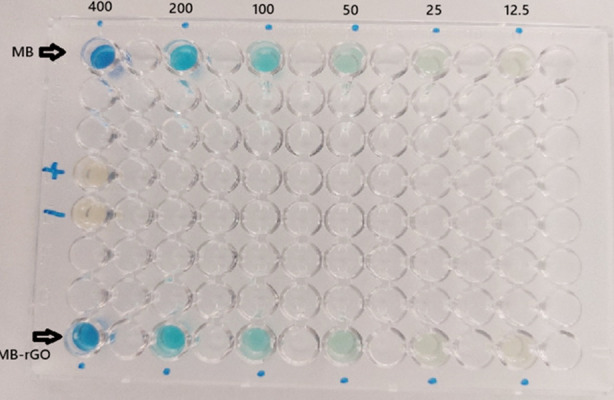
Layout of the 96-Well Microtiter Plate for Serial Dilution and Antibacterial Testing.

Experiments were conducted using 96-well polystyrene microtiter plates (BD Falcon, Franklin Lakes, NJ, USA). Four rows specifically rows 1, 4, 5, and 8—were designated for testing: laser-activated MB (1AL–6AL), purity check, sterility check, and laser-activated MB-rGO (1BL–6BL), respectively. Alternate wells were used to test different concentrations, as illustrated in Fig.2.

In rows one and eight, 100 µL of the respective stock solutions were added to the first and third wells. Subsequently, 100 µL of MHB was added to wells 3, 5, 7, 9, and 11. Serial dilutions were performed by transferring 100 µL from well to well, starting at well 3. Each well then received 100 µL of the bacterial suspension, producing final concentrations of 400, 200, 100, 50, 25, and 12.5 µg/mL. Purity and sterility control wells received 100 µL of bacterial suspension and MHB, respectively.

### The experimental layout included:

- **Laser-activated MB** (1AL–6AL)
- **Laser-activated MB-rGO** (1BL–6BL)

Photoactivation was carried out using a diode laser at ~660 nm, aligning with methylene blue’s peak absorption, for a duration of two minutes.^20^ Positive controls (untreated bacterial suspension) and negative controls (sterile broth) were included. All plates were incubated aerobically at 37°C for 24 hours, and sterility and purity controls were included in each experimental batch.

### MIC and MBC Determination:

The minimum inhibitory concentration (MIC) was defined as the lowest concentration at which no visible turbidity was observed after 24 hours of incubation at 37°C.^16^ For minimum bactericidal concentration (MBC) determination, 100 µL from wells showing no turbidity after 24 hours of incubation were sub-cultured onto SBA plates and spread using a sterile bent rod. Plates were incubated at 37°C for an additional 24 hours. The MBC was the lowest concentration that resulted in no visible colony formation.

All MIC and MBC tests were repeated three times using independently prepared bacterial suspensions and fresh dilutions on separate days to ensure consistency. The final MIC and MBC values were reported as means ± standard deviations. Intergroup comparisons were analyzed using the Wilcoxon signed-rank test. A *p*-value of ≤ 0.05 was considered statistically significant.

## RESULTS

**Fig 3:**
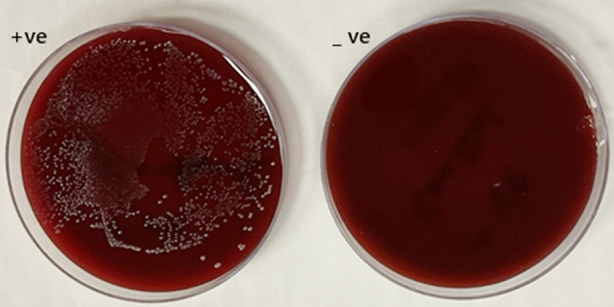
Images of Positive and Negative Control Wells Following Incubation.

**Fig 4:**
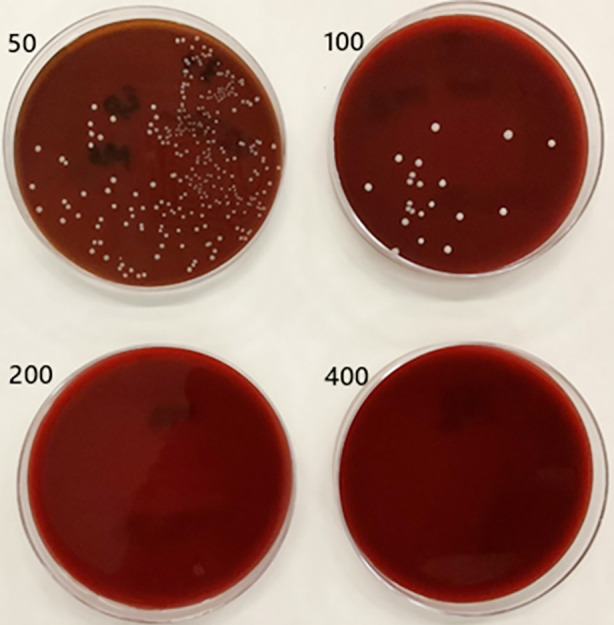
Spread Plate Results of MB Treatment Groups at Concentrations of 50, 100, 200, and 400 µg/mL Showing MIC at 50 µg/mL and MBC at 200 µg/mL.

**Fig 5:**
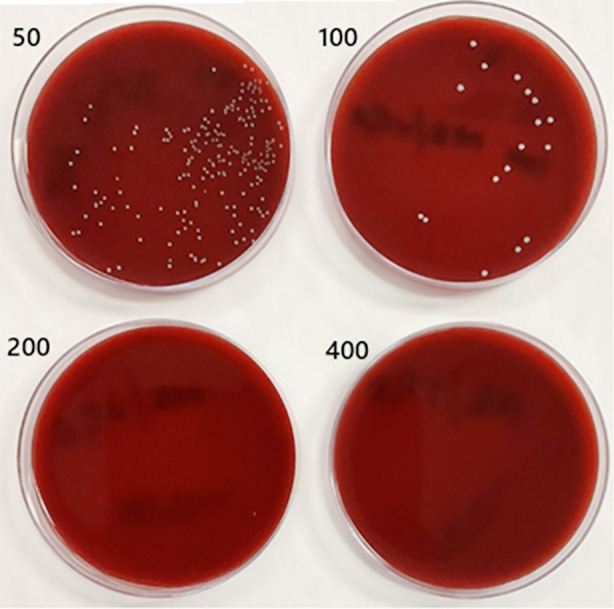
Spread Plate Results of MB-rGO Treatment Groups at Concentrations of 50, 100, 200, and 400 µg/mL Showing MIC at 50 µg/mL and MBC at 200 µg/mL.

Visible turbidity was observed in the MB group at concentrations of 12.5 µg/mL (6AL) and 25 µg/mL (5AL), and in the MB/rGO group at the same respective concentrations (6BL and 5BL). All higher concentrations in both groups exhibited no turbidity, indicating complete inhibition of microbial growth. The positive and negative wells were cultured with the spread method as shown in Fig.3. The minimum inhibitory concentration (MIC) and minimum bactericidal concentration (MBC) were determined using the spread plate method across three independent trials. For the MB group, the MIC averaged 66.67 ± 28.87 µg/mL, and the MBC was 266.67 ± 115.47 µg/mL. In contrast, the MB-rGO group demonstrated consistent values across all trials, with both MIC and MBC measured at 50.00 ± 0.00 µg/mL and 200.00 ± 0.00 µg/mL, respectively, as depicted in Table-I, Fig.4, and Fig.5.

**Table-I:** Minimum Inhibitory and Bactericidal Concentration (MIC & MBC) values of Methylene Blue (MB) and MB-Reduced Graphene Oxide (MB-rGO) against *Enterococcus faecalis* across three independent trials.

Trial No.	MB - MIC (µg/mL)	MB - MBC (µg/mL)	MB-rGO - MIC (µg/mL)	MB-rGO - MBC (µg/mL)
1	50	200	50	200
2	50	200	50	200
3	100	400	50	200
Mean ± SD	66.67 ± 28.87	266.67 ± 115.47	50.00 ± 0.00	200.00 ± 0.00

Under photoactivation, the comparison of MIC and MBC values between MB and MB-rGO against *E. faecalis* revealed no statistically significant differences. The mean MIC for MB was 66.67 ± 28.87 µg/mL, while that for MB-rGO was 50.00 ± 0.00 µg/mL (p = 0.317). Similarly, the MBC values were 266.67 ± 115.47 µg/mL for MB and 200.00 ± 0.00 µg/mL for MB-rGO (p = 0.317). Within-group comparisons showed that the MBC was higher than the MIC for both MB and MB-rGO, but the differences were statistically insignificant. For MB, the MIC vs. MBC comparison yielded a p-value of 0.102, while for MB-rGO, the MIC vs. MBC comparison resulted in a p-value of 0.083, as depicted in Table-II.

**Table-II:** Comparative Statistical Analysis of MIC and MBC Values of MB and MB-rGO Against *Enterococcus faecalis* Under Photoactivation.

Trial No.	Mean ± SD (µg/mL)	p-value
MIC (MB vs MB-rGO)	66.67 ± 28.87 vs 50.00 ± 0.00	0.317
MBC (MB vs MB-rGO)	266.67 ± 115.47 vs 200.00 ± 0.00	0.317
MB - MIC vs MB - MBC	66.67 ± 28.87 vs 266.67 ± 115.47	0.102
MB-rGO - MIC vs MB-rGO - MBC	50.00 ± 0.00 vs 200.00 ± 0.00	0.083

## DISCUSSION

This study investigated the antimicrobial efficacy of Methylene blue alone and reduced graphene oxide functionalized with methylene blue (MB-rGO) against *E. faecalis* using a broth microdilution method with photoactivation. The results demonstrated that both MB and MB-rGO exhibited effective antibacterial activity, with complete inhibition of microbial growth at concentrations ≥50 µg/mL. however, the MB-rGO group showed a lower and more consistent MIC and MBC (50.00 ± 0.00 µg/mL and 200.00 ± 0.00 µg/mL, respectively) compared to the MB-only group (66.67 ± 28.87 µg/mL and 266.67 ± 115.47 µg/mL), indicating enhanced and stable antimicrobial performance of the functionalized compound. Hence, the working hypothesis of the current study was partially accepted.

The enhanced efficacy of MB-rGO could be attributed to the synergistic effect between the photosensitizer, like MB and the graphene oxide platform, which potentially increases cellular uptake and reactive oxygen species (ROS) generation upon laser activation. These findings are consistent with prior literature supporting the role of graphene-based materials in improving aPDT outcomes.^21^,^22^

The present study demonstrated that laser-activated MB-rGO achieved comparable antimicrobial efficacy against *E. faecalis* at significantly lower concentrations, with MIC and MBC values of 50.00 ± 0.00 µg/mL and 200.00 ± 0.00 µg/mL, respectively. These findings show that laser-activated MB-rGO can exert strong antibacterial effects at significantly lower concentrations compared to silver nanoparticles, potentially minimizing cytotoxicity.^23^ In contrast, a prior study assessed the MIC and MBC of 5 nm silver nanoparticles (AgNPs) against *Staphylococcus aureus* using the macrodilution process, with both values reported at 625 µg/mL. Silver nanoparticles are often incorporated in the root canal medicaments, sealers and irrigants due to their potent antimicrobial efficacy against *S. aureus*.^24^ Similarly, another study found that the MIC and MBC of 10 nm silver nanoparticles against *S. aureus* were 1350 µg/mL^23^, which are significantly higher than the concentrations of MB-rGO used in the present study. This indicates that MB-rGO can achieve comparable or superior antimicrobial effects at much lower doses.

Additionally, in the present study, MB-rGO employed a light-activated mechanism to generate cytotoxic ROS, offering the advantages of targeted antimicrobial therapy with the potential for reduced systemic toxicity. In contrast, AgNPs have been recognized as broad-spectrum biocides effective against a wide range of drug-resistant pathogens, including *E. faecalis* and *S. aureus*.^25^,^26^ However, their cytotoxicity and long-term safety remain a concern.

The present study confirms that photodynamic therapy (PDT) can successfully combat *E. faecalis*. When laser-activated MB-rGO exhibited robust antimicrobial properties, achieving a consistent MIC of 50 ± 0 µg/mL and an MBC of 200 ± 0 µg/mL, evidence that it suppresses bacterial growth effectively even at comparatively low doses. Similarly, prior studies have reported significant reductions in *E. faecalis* counts using various PDT approaches. For instance, methylene blue-mediated PDT (MBaPDT) exhibited superior antibacterial effects compared to control groups, as observed in studies by Lopez-Jimenez et al., where MB/670 nm aPDT significantly disrupted *E. faecalis* biofilms.^7^ Likewise, Beltes et al. and Akbari et al. demonstrated effective bacterial reduction using indocyanine green (ICG)-based PDT systems, with the latter reporting enhanced efficacy when ICG was ionized into nanographene oxide (ICGnGO), highlighting the role of graphene-based photosensitizers in improving photodynamic outcomes.^27^,^28^

Interestingly, another study evaluated the effects of MB, MBaPDT, ICG, and dual-dye aPDT against oral biofilms formed by various bacteria such as *S. mutans*, *E. faecalis*, and *P. intermedia*. The result of the study revealed a signification drop in *E. faecalis* counts by MBaPDT compared to MB alone and ICG. This highlights the MB’s strong photodynamic effect against this pathogen. While dual-dye aPDT showed some antibacterial activity against *E. faecalis*, but showed promising results against other bacteria. 29 In contrast, the present research focused exclusively on *E. faecalis* and enhanced the antibacterial potential of MB by functionalizing it with reduced graphene oxide, resulting in highly reproducible and concentration-efficient bacterial inhibition.

### Strength of the study:

This study’s strength lies in its use of a standardized bacterial strain (*E. faecalis* ATCC 29212) and validated broth microdilution methods following CLSI guidelines, ensuring reproducibility and reliability of results. The functionalization of methylene blue with reduced graphene oxide introduces a novel and promising approach for enhancing antimicrobial efficacy in root canal disinfection. Additionally, the incorporation of laser activation mimics clinical photodynamic therapy conditions, improving the translational relevance of the findings. Triplicate testing and consistent methodology further strengthen the robustness of the data.

### Limitations:

This study was limited by a small number of experimental trials and the use of a single bacterial strain (*E. faecalis*), which may not fully represent broader microbial responses. Additionally, in vitro conditions may not accurately reflect clinical performance.

## CONCLUSION

MB-rGO demonstrated more consistent and lower MIC and MBC values than MB alone, indicating stronger and more reliable antimicrobial activity against *E. faecalis*. The absence of turbidity at higher concentrations further supports its potential as an effective antibacterial agent. Overall, both agents effectively inhibited microbial growth at higher concentrations, with no turbidity observed, indicating their potential utility in photodynamic antimicrobial therapy.

### Recommandations:

Future studies should include a larger sample size, multiple bacterial species, and in vivo models to validate the antimicrobial efficacy of MB-rGO. Exploring long-term stability and potential cytotoxicity is also recommended.

### Authors Contribution:

**RJ and MSH:** Concept and accountable for the accuracy of the study.

**RJ:** Literature Search, Writing.

**RJ, SAQ and MSH:** Data Collection and Processing. Analysis and Interpretation

**MSH, SAQ and MAA:** Review,

**MSH and MAA:** Supervision.

All authors have critically reviewed and approved the final draft.
